# Methyl 5-(4-acet­oxy­phen­yl)-2-(2-bromo­benzyl­idine)-7-methyl-3-oxo-2,3-di­hydro-5*H*-1,3-thia­zolo[3,2-*a*]pyrimidine-6-carboxyl­ate

**DOI:** 10.1107/S1600536813019132

**Published:** 2013-07-13

**Authors:** Nikhath Fathima, H. Nagarajaiah, Noor Shahina Begum

**Affiliations:** a Department of Chemistry, Bangalore University, Bangalore 560 001, India

## Abstract

In the title mol­ecule, C_24_H_19_BrN_2_O_5_S, the pyrimidine ring is in a flattened half-chair conformation and the 4-acet­oxy­phenyl group is substituted axially to this ring. The thia­zole ring is essentially planar [with a maximum deviation of 0.012 (2) Å for the N atom] and forms dihedral angles of 17.65 (13) and 88.95 (11)° with the bromo- and acet­oxy-substituted benzene rings, respectively. The dihedral angle between the benzene rings is 81.84 (13) Å. In the crystal, pairs of weak C—H⋯O hydrogen bonds lead to the formation of inversion dimers. A weak C—H⋯π inter­action and π–π stacking inter­actions with centroid–centroid distances of 3.5903 (14) Å are observed.

## Related literature

For the biological activity of di­hydro­pyrimidines, see: Alam *et al.* (2010 ▶); Kappe (2000 ▶); Atwal *et al.* (1991 ▶); Rovnyak *et al.* (1992 ▶). For related structures, see: Nagarajaiah *et al.* (2011 ▶, 2012 ▶). For hydrogen-bond graph-set motifs, see: Bernstein *et al.* (1995 ▶).
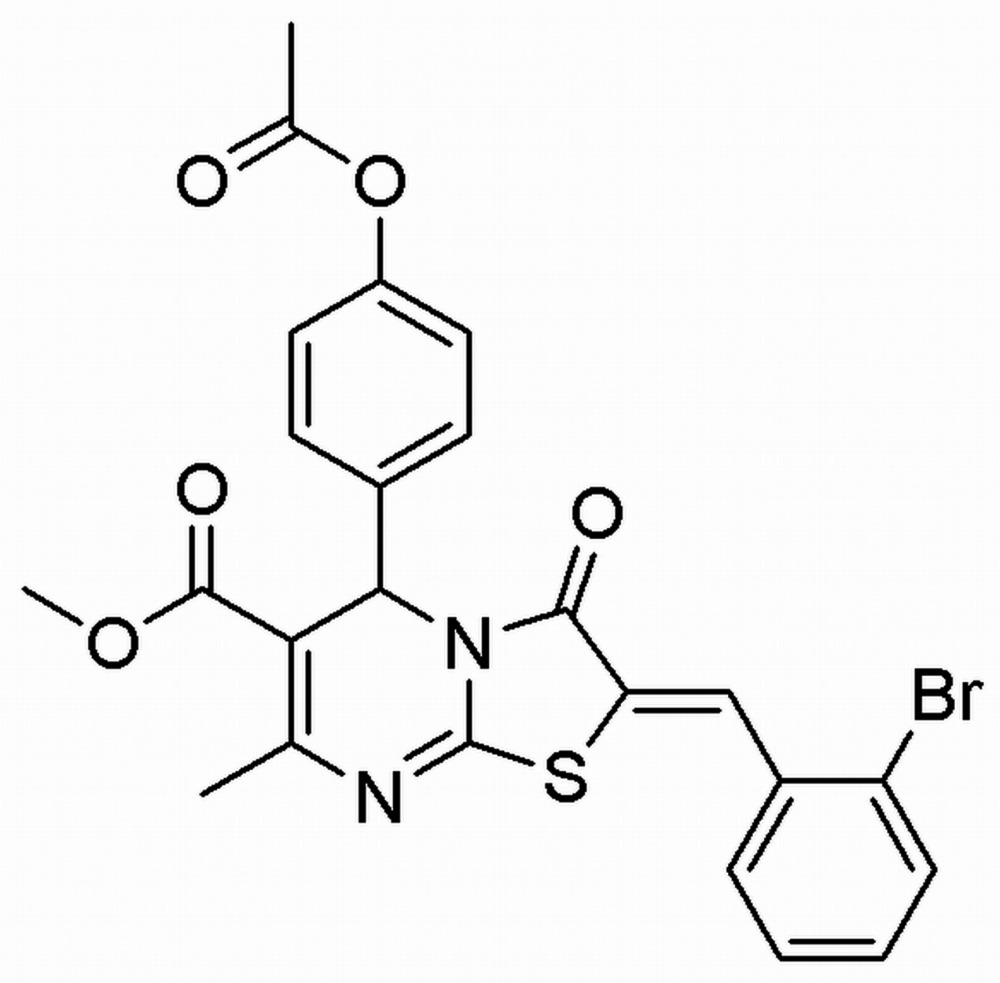


## Experimental

### 

#### Crystal data

C_24_H_19_BrN_2_O_5_S
*M* *_r_* = 527.38
Triclinic, 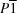

*a* = 7.6018 (5) Å
*b* = 11.9648 (7) Å
*c* = 14.0877 (9) Å
α = 106.425 (1)°
β = 104.700 (2)°
γ = 106.296 (1)°
*V* = 1099.75 (12) Å^3^
*Z* = 2
Mo *K*α radiation
μ = 2.00 mm^−1^
*T* = 296 K
0.18 × 0.16 × 0.16 mm

#### Data collection

Bruker SMART APEX CCD detector diffractometer
Absorption correction: multi-scan (*SADABS*; Bruker, 1998 ▶) *T*_min_ = 0.714, *T*_max_ = 0.740
9029 measured reflections
4777 independent reflections
3989 reflections with *I* > 2σ(*I*)
*R*_int_ = 0.026

#### Refinement

*R*[*F* ^2^ > 2σ(*F* ^2^)] = 0.039
*wR*(*F* ^2^) = 0.096
*S* = 1.05
4777 reflections
301 parameters
H-atom parameters constrained
Δρ_max_ = 0.58 e Å^−3^
Δρ_min_ = −0.61 e Å^−3^

### 

Data collection: *SMART* (Bruker, 1998 ▶); cell refinement: *SAINT-Plus* (Bruker, 1998 ▶); data reduction: *SAINT-Plus*; program(s) used to solve structure: *SHELXS97* (Sheldrick, 2008 ▶); program(s) used to refine structure: *SHELXL97* (Sheldrick, 2008 ▶); molecular graphics: *ORTEP-3 for Windows* (Farrugia, 2012 ▶) and *CAMERON* (Watkin *et al.*, 1996 ▶); software used to prepare material for publication: *WinGX* (Farrugia, 2012 ▶).

## Supplementary Material

Crystal structure: contains datablock(s) global, I. DOI: 10.1107/S1600536813019132/lh5628sup1.cif

Structure factors: contains datablock(s) I. DOI: 10.1107/S1600536813019132/lh5628Isup2.hkl

Additional supplementary materials:  crystallographic information; 3D view; checkCIF report

## Figures and Tables

**Table 1:** Hydrogen-bond geometry (Å, °) *Cg* is the centroid of the C5–C7/C9/N1/N2 ring.

*D*—H⋯*A*	*D*—H	H⋯*A*	*D*⋯*A*	*D*—H⋯*A*
C13—H13⋯O1^i^	0.93	2.60	3.343 (4)	138
C10—H10⋯*Cg* ^ii^	0.93	2.61	3.513 (4)	147

Symmetry codes: (i) 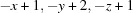; (ii) 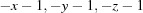.

**Fig. 1.**
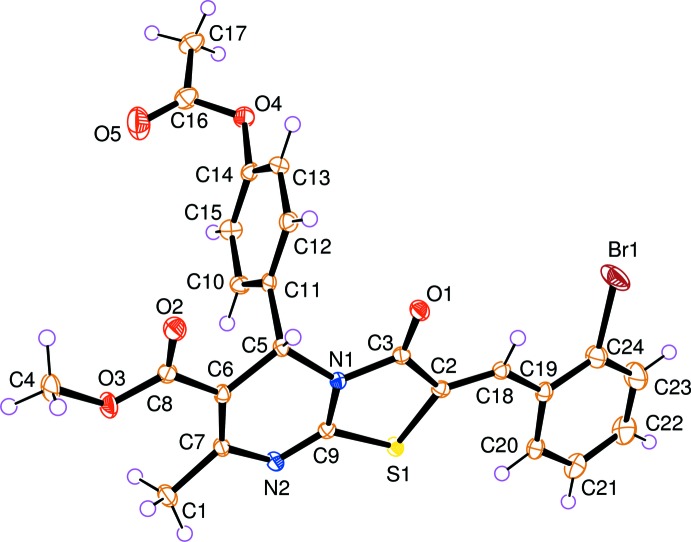
The molecular structure of the title compound, showing 50% probability ellipsoids.

**Fig. 2.**
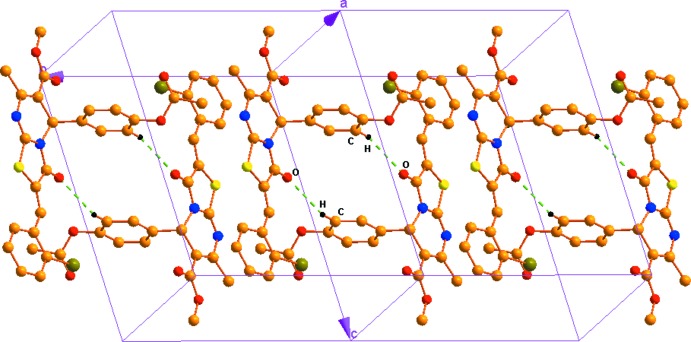
The packing of the title compound showing weak C—H···O hydrogen bonds with dashed lines. H-atoms not involved in hydrogen bonding have been excluded.

## supplementary crystallographic information

### Comment

Dihydropyrimidines (DHPM) exhibit a broad range of therapeutic and pharmacological properties (Alam *et al.*, 2010). These non-planar heterocyclic compounds have interesting multifaceted pharmacological profiles such as calcium channel modulation, antitumor, antiviral, antibacterial, anti-inflammatory and antimicrobial activities (Kappe *et al.*, 2000; Atwal *et al.*. 1991). In addition, a few DHPM derivatives have even emerged as orally active antihypertensive agents (Rovnyak *et al.*, 1992).

The molecular structure of the title compound is shown in Fig. 1. The 4-acetoxy substituted benzene ring is axially substituted to the pyrimidine ring. The central pyrimidine ring adopts a half chair conformation with deviations of 0.107 (2) and -0.200 (2) Å for N1 and C5 respectively from the remaining four ring atoms (C6/C7/C9/N2). In the crystal, pairs of weak C—H···O hydrogen bonds form inversion dimers with a graph set notation of *R*^2^_2_(16) (Bernstein *et al.*, 1995) as shown in Fig. 2. The crystal structure of the title compound shows different type of intermolecular interactions compared to a similar structure reported earlier (Nagarajaiah *et al.*, 2012). In addition, there are intermolecular π···π interactions between inversion-related thiazole rings with a centroid to centroid distance of 3.5903 (14)Å. A weak C—H···π(ring) interaction is also observed (see Table 1, where *Cg* is the centroid of the C5/C6/C7/N2/C9/N1 ring). Another example of a related crystal structure has been published in the literature (Nagarajaiah *et al.*, 2011).

### Experimental

The compound 2-(2-bromo-benzylidene)-4-(4-hydroxy-phenyl)6-methyl-3-oxo-2,3-dihydro-thieno[2,3-bpyridine-5-carboxylic acid (2.0 g) was mixed with acetic anhydride (10 ml) and refluxed for about 4 h. The reaction mixture was cooled and diluted by the addition of water (20 ml). The solid separated was washed with water, filtered, and dried (Yield: 2.63 g, 80% and mp 418 K). Pale yellow crystals suitable for diffraction were obtained by slow evaporation of a solution of the title compound in chloroform.

### Refinement

The H atoms were placed in calculated positions in a riding-model approximation with C—H = 0.93 Å, 0.96 Å and 0.98 Å for aryl, methyl and methyne H-atoms respectively, with *U*_iso_(H) = 1.5*U*_eq_(C) for methyl H atoms and *U*_iso_(H) = 1.2*U*_eq_(C) for other H atoms.

### Crystal data

**Table:**

C_24_H_19_BrN_2_O_5_S	*Z* = 2
*M**_r_* = 527.38	*F*(000) = 536
Triclinic, *P*1	*D*_x_ = 1.593 Mg m^−^^3^
Hall symbol: -P 1	Mo *K*α radiation, λ = 0.71073 Å
*a* = 7.6018 (5) Å	Cell parameters from 4777 reflections
*b* = 11.9648 (7) Å	θ = 2.8–27.0°
*c* = 14.0877 (9) Å	µ = 2.00 mm^−^^1^
α = 106.425 (1)°	*T* = 296 K
β = 104.700 (2)°	Block, yellow
γ = 106.296 (1)°	0.18 × 0.16 × 0.16 mm
*V* = 1099.75 (12) Å^3^	

### Data collection

**Table:**

Bruker SMART APEX CCD detector diffractometer	4777 independent reflections
Radiation source: fine-focus sealed tube	3989 reflections with *I* > 2σ(*I*)
Graphite monochromator	*R*_int_ = 0.026
ω scans	θ_max_ = 27.0°, θ_min_ = 2.8°
Absorption correction: multi-scan (*SADABS*; Bruker, 1998)	*h* = −7→9
*T*_min_ = 0.714, *T*_max_ = 0.740	*k* = −15→15
9029 measured reflections	*l* = −17→16

### Refinement

**Table:**

Refinement on *F*^2^	Primary atom site location: structure-invariant direct methods
Least-squares matrix: full	Secondary atom site location: difference Fourier map
*R*[*F*^2^ > 2σ(*F*^2^)] = 0.039	Hydrogen site location: inferred from neighbouring sites
*wR*(*F*^2^) = 0.096	H-atom parameters constrained
*S* = 1.05	*w* = 1/[σ^2^(*F*_o_^2^) + (0.0443*P*)^2^ + 0.7937*P*] where *P* = (*F*_o_^2^ + 2*F*_c_^2^)/3
4777 reflections	(Δ/σ)_max_ < 0.001
301 parameters	Δρ_max_ = 0.58 e Å^−^^3^
0 restraints	Δρ_min_ = −0.61 e Å^−^^3^

### Special details

**Table:**

Geometry. All s.u.'s (except the s.u. in the dihedral angle between two l.s. planes) are estimated using the full covariance matrix. The cell s.u.'s are taken into account individually in the estimation of s.u.'s in distances, angles and torsion angles; correlations between s.u.'s in cell parameters are only used when they are defined by crystal symmetry. An approximate (isotropic) treatment of cell s.u.'s is used for estimating s.u.'s involving l.s. planes.
Refinement. Refinement of *F*^2^ against ALL reflections. The weighted *R*-factor *wR* and goodness of fit *S* are based on *F*^2^, conventional *R*-factors *R* are based on *F*, with *F* set to zero for negative *F*^2^. The threshold expression of *F*^2^ > 2σ(*F*^2^) is used only for calculating *R*-factors(gt) *etc*. and is not relevant to the choice of reflections for refinement. *R*-factors based on *F*^2^ are statistically about twice as large as those based on *F*, and *R*- factors based on ALL data will be even larger.

### Fractional atomic coordinates and isotropic or equivalent isotropic displacement parameters (Å^2^)

**Table:**

	*x*	*y*	*z*	*U*_iso_*/*U*_eq_	
C1	0.3032 (4)	0.5765 (2)	0.84902 (18)	0.0216 (5)	
H1A	0.4212	0.6045	0.9093	0.032*	
H1B	0.2507	0.4862	0.8164	0.032*	
H1C	0.2083	0.6040	0.8716	0.032*	
C2	0.3542 (3)	0.5692 (2)	0.42420 (17)	0.0137 (4)	
C3	0.4784 (3)	0.6889 (2)	0.51728 (17)	0.0135 (4)	
C4	0.6434 (4)	0.9217 (2)	1.08072 (18)	0.0294 (6)	
H4A	0.7732	0.9210	1.1031	0.044*	
H4B	0.5811	0.9043	1.1291	0.044*	
H4C	0.6517	1.0030	1.0800	0.044*	
C5	0.5037 (3)	0.7934 (2)	0.70450 (16)	0.0125 (4)	
H5	0.6450	0.8395	0.7254	0.015*	
C6	0.4688 (3)	0.7494 (2)	0.79134 (17)	0.0139 (4)	
C7	0.3495 (3)	0.6308 (2)	0.77040 (17)	0.0141 (4)	
C8	0.5830 (3)	0.8511 (2)	0.89783 (17)	0.0164 (5)	
C9	0.3027 (3)	0.5690 (2)	0.59347 (17)	0.0129 (4)	
C10	0.2005 (3)	0.8465 (2)	0.67420 (17)	0.0164 (5)	
H10	0.1380	0.7744	0.6844	0.020*	
C11	0.3962 (3)	0.8789 (2)	0.68289 (16)	0.0129 (4)	
C12	0.4888 (3)	0.9866 (2)	0.66788 (18)	0.0169 (5)	
H12	0.6196	1.0090	0.6737	0.020*	
C13	0.3871 (4)	1.0614 (2)	0.64419 (18)	0.0196 (5)	
H13	0.4489	1.1333	0.6336	0.024*	
C14	0.1933 (4)	1.0274 (2)	0.63663 (17)	0.0177 (5)	
C15	0.0973 (3)	0.9204 (2)	0.65043 (19)	0.0190 (5)	
H15	−0.0339	0.8980	0.6440	0.023*	
C16	0.0214 (4)	1.1615 (2)	0.6767 (2)	0.0237 (5)	
C17	−0.0856 (4)	1.2323 (2)	0.6298 (2)	0.0259 (6)	
H17A	−0.1463	1.2671	0.6763	0.039*	
H17B	−0.1848	1.1760	0.5620	0.039*	
H17C	0.0057	1.2991	0.6213	0.039*	
C18	0.3621 (3)	0.5584 (2)	0.32793 (17)	0.0145 (4)	
H18	0.4543	0.6275	0.3266	0.017*	
C19	0.2483 (3)	0.4550 (2)	0.22544 (18)	0.0163 (5)	
C20	0.1439 (3)	0.3325 (2)	0.21514 (19)	0.0180 (5)	
H20	0.1464	0.3160	0.2760	0.022*	
C21	0.0370 (4)	0.2353 (2)	0.1167 (2)	0.0244 (5)	
H21	−0.0314	0.1550	0.1121	0.029*	
C22	0.0323 (4)	0.2580 (3)	0.0253 (2)	0.0322 (6)	
H22	−0.0401	0.1931	−0.0408	0.039*	
C23	0.1350 (5)	0.3770 (3)	0.0321 (2)	0.0355 (7)	
H23	0.1331	0.3922	−0.0293	0.043*	
C24	0.2407 (4)	0.4736 (2)	0.13075 (19)	0.0257 (6)	
Br1	0.37745 (6)	0.63467 (3)	0.13393 (2)	0.04578 (13)	
N1	0.4376 (3)	0.68110 (17)	0.60657 (14)	0.0122 (4)	
N2	0.2509 (3)	0.53974 (17)	0.66529 (14)	0.0150 (4)	
O1	0.5953 (2)	0.78204 (15)	0.51788 (12)	0.0176 (3)	
O2	0.7097 (2)	0.94816 (15)	0.91182 (13)	0.0216 (4)	
O3	0.5290 (3)	0.82642 (16)	0.97517 (13)	0.0251 (4)	
O4	0.0946 (3)	1.10282 (16)	0.60848 (13)	0.0243 (4)	
O5	0.0390 (4)	1.1543 (2)	0.76119 (17)	0.0451 (6)	
S1	0.20567 (8)	0.46190 (5)	0.46166 (4)	0.01442 (13)	

### Atomic displacement parameters (Å^2^)

**Table:**

	*U*^11^	*U*^22^	*U*^33^	*U*^12^	*U*^13^	*U*^23^
C1	0.0278 (13)	0.0208 (12)	0.0168 (11)	0.0060 (10)	0.0100 (10)	0.0095 (9)
C2	0.0122 (11)	0.0147 (10)	0.0162 (10)	0.0067 (9)	0.0051 (9)	0.0073 (9)
C3	0.0142 (11)	0.0164 (11)	0.0136 (10)	0.0087 (9)	0.0055 (8)	0.0076 (9)
C4	0.0452 (17)	0.0241 (13)	0.0095 (11)	0.0075 (12)	0.0061 (11)	0.0022 (10)
C5	0.0119 (10)	0.0130 (10)	0.0105 (9)	0.0036 (8)	0.0030 (8)	0.0036 (8)
C6	0.0152 (11)	0.0178 (11)	0.0109 (10)	0.0093 (9)	0.0047 (8)	0.0055 (9)
C7	0.0158 (11)	0.0173 (11)	0.0124 (10)	0.0081 (9)	0.0065 (9)	0.0070 (9)
C8	0.0202 (12)	0.0189 (11)	0.0129 (10)	0.0118 (10)	0.0047 (9)	0.0067 (9)
C9	0.0119 (10)	0.0137 (10)	0.0130 (10)	0.0062 (9)	0.0031 (8)	0.0051 (8)
C10	0.0162 (11)	0.0138 (10)	0.0167 (11)	0.0028 (9)	0.0054 (9)	0.0057 (9)
C11	0.0150 (11)	0.0131 (10)	0.0081 (9)	0.0044 (9)	0.0032 (8)	0.0021 (8)
C12	0.0165 (11)	0.0170 (11)	0.0180 (11)	0.0059 (9)	0.0087 (9)	0.0061 (9)
C13	0.0283 (13)	0.0149 (11)	0.0195 (11)	0.0085 (10)	0.0119 (10)	0.0090 (9)
C14	0.0257 (13)	0.0187 (11)	0.0119 (10)	0.0139 (10)	0.0058 (9)	0.0055 (9)
C15	0.0138 (11)	0.0199 (11)	0.0216 (11)	0.0062 (9)	0.0040 (9)	0.0079 (9)
C16	0.0229 (13)	0.0229 (12)	0.0249 (13)	0.0114 (11)	0.0065 (10)	0.0075 (10)
C17	0.0267 (14)	0.0229 (13)	0.0294 (13)	0.0148 (11)	0.0075 (11)	0.0085 (11)
C18	0.0148 (11)	0.0145 (10)	0.0152 (10)	0.0067 (9)	0.0055 (9)	0.0058 (9)
C19	0.0153 (11)	0.0206 (11)	0.0150 (10)	0.0100 (10)	0.0059 (9)	0.0061 (9)
C20	0.0148 (11)	0.0212 (12)	0.0186 (11)	0.0080 (10)	0.0076 (9)	0.0060 (9)
C21	0.0181 (12)	0.0206 (12)	0.0249 (13)	0.0029 (10)	0.0067 (10)	0.0005 (10)
C22	0.0327 (15)	0.0285 (14)	0.0182 (12)	0.0047 (12)	0.0028 (11)	−0.0027 (11)
C23	0.0506 (18)	0.0353 (16)	0.0148 (12)	0.0130 (14)	0.0091 (12)	0.0072 (11)
C24	0.0346 (15)	0.0217 (12)	0.0172 (12)	0.0082 (11)	0.0081 (11)	0.0059 (10)
Br1	0.0837 (3)	0.02598 (16)	0.01761 (15)	0.00523 (15)	0.01693 (15)	0.01100 (11)
N1	0.0133 (9)	0.0127 (9)	0.0109 (8)	0.0053 (7)	0.0046 (7)	0.0046 (7)
N2	0.0154 (9)	0.0158 (9)	0.0133 (9)	0.0043 (8)	0.0053 (7)	0.0063 (7)
O1	0.0202 (8)	0.0156 (8)	0.0158 (8)	0.0038 (7)	0.0076 (7)	0.0063 (6)
O2	0.0247 (9)	0.0173 (8)	0.0163 (8)	0.0032 (7)	0.0052 (7)	0.0041 (7)
O3	0.0368 (11)	0.0193 (8)	0.0110 (8)	0.0030 (8)	0.0080 (7)	0.0027 (7)
O4	0.0357 (10)	0.0278 (9)	0.0220 (9)	0.0226 (8)	0.0133 (8)	0.0143 (7)
O5	0.0676 (16)	0.0675 (16)	0.0336 (11)	0.0522 (14)	0.0300 (11)	0.0293 (11)
S1	0.0143 (3)	0.0142 (3)	0.0112 (2)	0.0025 (2)	0.0034 (2)	0.0038 (2)

### Geometric parameters (Å, º)

**Table:**

C1—C7	1.500 (3)	C11—C12	1.385 (3)
C1—H1A	0.9600	C12—C13	1.391 (3)
C1—H1B	0.9600	C12—H12	0.9300
C1—H1C	0.9600	C13—C14	1.381 (4)
C2—C18	1.344 (3)	C13—H13	0.9300
C2—C3	1.487 (3)	C14—C15	1.378 (3)
C2—S1	1.754 (2)	C14—O4	1.402 (3)
C3—O1	1.211 (3)	C15—H15	0.9300
C3—N1	1.391 (3)	C16—O5	1.195 (3)
C4—O3	1.448 (3)	C16—O4	1.354 (3)
C4—H4A	0.9600	C16—C17	1.497 (3)
C4—H4B	0.9600	C17—H17A	0.9600
C4—H4C	0.9600	C17—H17B	0.9600
C5—N1	1.478 (3)	C17—H17C	0.9600
C5—C6	1.515 (3)	C18—C19	1.456 (3)
C5—C11	1.525 (3)	C18—H18	0.9300
C5—H5	0.9800	C19—C24	1.403 (3)
C6—C7	1.352 (3)	C19—C20	1.403 (3)
C6—C8	1.483 (3)	C20—C21	1.387 (3)
C7—N2	1.417 (3)	C20—H20	0.9300
C8—O2	1.209 (3)	C21—C22	1.382 (4)
C8—O3	1.335 (3)	C21—H21	0.9300
C9—N2	1.275 (3)	C22—C23	1.381 (4)
C9—N1	1.370 (3)	C22—H22	0.9300
C9—S1	1.750 (2)	C23—C24	1.384 (4)
C10—C15	1.391 (3)	C23—H23	0.9300
C10—C11	1.391 (3)	C24—Br1	1.898 (3)
C10—H10	0.9300		
			
C7—C1—H1A	109.5	C14—C13—H13	120.4
C7—C1—H1B	109.5	C12—C13—H13	120.4
H1A—C1—H1B	109.5	C15—C14—C13	121.7 (2)
C7—C1—H1C	109.5	C15—C14—O4	121.2 (2)
H1A—C1—H1C	109.5	C13—C14—O4	117.1 (2)
H1B—C1—H1C	109.5	C14—C15—C10	118.7 (2)
C18—C2—C3	119.9 (2)	C14—C15—H15	120.7
C18—C2—S1	129.72 (18)	C10—C15—H15	120.7
C3—C2—S1	110.36 (16)	O5—C16—O4	123.4 (2)
O1—C3—N1	123.4 (2)	O5—C16—C17	126.0 (2)
O1—C3—C2	126.8 (2)	O4—C16—C17	110.6 (2)
N1—C3—C2	109.76 (19)	C16—C17—H17A	109.5
O3—C4—H4A	109.5	C16—C17—H17B	109.5
O3—C4—H4B	109.5	H17A—C17—H17B	109.5
H4A—C4—H4B	109.5	C16—C17—H17C	109.5
O3—C4—H4C	109.5	H17A—C17—H17C	109.5
H4A—C4—H4C	109.5	H17B—C17—H17C	109.5
H4B—C4—H4C	109.5	C2—C18—C19	129.8 (2)
N1—C5—C6	108.19 (17)	C2—C18—H18	115.1
N1—C5—C11	109.45 (16)	C19—C18—H18	115.1
C6—C5—C11	113.06 (18)	C24—C19—C20	116.4 (2)
N1—C5—H5	108.7	C24—C19—C18	120.8 (2)
C6—C5—H5	108.7	C20—C19—C18	122.8 (2)
C11—C5—H5	108.7	C21—C20—C19	121.8 (2)
C7—C6—C8	126.3 (2)	C21—C20—H20	119.1
C7—C6—C5	122.34 (19)	C19—C20—H20	119.1
C8—C6—C5	111.35 (19)	C22—C21—C20	119.9 (2)
C6—C7—N2	121.6 (2)	C22—C21—H21	120.1
C6—C7—C1	127.3 (2)	C20—C21—H21	120.1
N2—C7—C1	111.08 (19)	C23—C22—C21	120.1 (2)
O2—C8—O3	123.7 (2)	C23—C22—H22	119.9
O2—C8—C6	122.5 (2)	C21—C22—H22	119.9
O3—C8—C6	113.8 (2)	C22—C23—C24	119.6 (3)
N2—C9—N1	126.87 (19)	C22—C23—H23	120.2
N2—C9—S1	121.52 (17)	C24—C23—H23	120.2
N1—C9—S1	111.59 (16)	C23—C24—C19	122.2 (2)
C15—C10—C11	120.8 (2)	C23—C24—Br1	117.3 (2)
C15—C10—H10	119.6	C19—C24—Br1	120.52 (18)
C11—C10—H10	119.6	C9—N1—C3	116.59 (18)
C12—C11—C10	119.3 (2)	C9—N1—C5	120.26 (18)
C12—C11—C5	120.8 (2)	C3—N1—C5	122.05 (18)
C10—C11—C5	119.8 (2)	C9—N2—C7	116.48 (19)
C11—C12—C13	120.4 (2)	C8—O3—C4	115.0 (2)
C11—C12—H12	119.8	C16—O4—C14	118.97 (19)
C13—C12—H12	119.8	C9—S1—C2	91.67 (11)
C14—C13—C12	119.1 (2)		
			
C18—C2—C3—O1	−1.9 (4)	C19—C20—C21—C22	0.3 (4)
S1—C2—C3—O1	−179.35 (19)	C20—C21—C22—C23	0.5 (4)
C18—C2—C3—N1	176.6 (2)	C21—C22—C23—C24	−0.7 (5)
S1—C2—C3—N1	−0.9 (2)	C22—C23—C24—C19	0.2 (5)
N1—C5—C6—C7	−16.1 (3)	C22—C23—C24—Br1	−179.8 (2)
C11—C5—C6—C7	105.3 (2)	C20—C19—C24—C23	0.5 (4)
N1—C5—C6—C8	162.92 (17)	C18—C19—C24—C23	179.5 (3)
C11—C5—C6—C8	−75.7 (2)	C20—C19—C24—Br1	−179.49 (17)
C8—C6—C7—N2	−178.3 (2)	C18—C19—C24—Br1	−0.4 (3)
C5—C6—C7—N2	0.6 (3)	N2—C9—N1—C3	176.5 (2)
C8—C6—C7—C1	1.2 (4)	S1—C9—N1—C3	−2.1 (2)
C5—C6—C7—C1	−179.9 (2)	N2—C9—N1—C5	−15.3 (3)
C7—C6—C8—O2	168.1 (2)	S1—C9—N1—C5	166.10 (15)
C5—C6—C8—O2	−10.9 (3)	O1—C3—N1—C9	−179.5 (2)
C7—C6—C8—O3	−13.6 (3)	C2—C3—N1—C9	2.0 (3)
C5—C6—C8—O3	167.38 (18)	O1—C3—N1—C5	12.5 (3)
C15—C10—C11—C12	0.2 (3)	C2—C3—N1—C5	−166.05 (18)
C15—C10—C11—C5	−177.5 (2)	C6—C5—N1—C9	22.8 (3)
N1—C5—C11—C12	−102.4 (2)	C11—C5—N1—C9	−100.8 (2)
C6—C5—C11—C12	137.0 (2)	C6—C5—N1—C3	−169.64 (18)
N1—C5—C11—C10	75.3 (2)	C11—C5—N1—C3	66.8 (2)
C6—C5—C11—C10	−45.4 (3)	N1—C9—N2—C7	−2.9 (3)
C10—C11—C12—C13	−0.1 (3)	S1—C9—N2—C7	175.63 (15)
C5—C11—C12—C13	177.53 (19)	C6—C7—N2—C9	10.2 (3)
C11—C12—C13—C14	0.5 (3)	C1—C7—N2—C9	−169.4 (2)
C12—C13—C14—C15	−1.0 (3)	O2—C8—O3—C4	−4.2 (3)
C12—C13—C14—O4	−177.54 (19)	C6—C8—O3—C4	177.6 (2)
C13—C14—C15—C10	1.0 (3)	O5—C16—O4—C14	1.3 (4)
O4—C14—C15—C10	177.5 (2)	C17—C16—O4—C14	−177.5 (2)
C11—C10—C15—C14	−0.6 (3)	C15—C14—O4—C16	63.7 (3)
C3—C2—C18—C19	−176.1 (2)	C13—C14—O4—C16	−119.7 (2)
S1—C2—C18—C19	0.8 (4)	N2—C9—S1—C2	−177.4 (2)
C2—C18—C19—C24	163.0 (2)	N1—C9—S1—C2	1.26 (17)
C2—C18—C19—C20	−18.0 (4)	C18—C2—S1—C9	−177.3 (2)
C24—C19—C20—C21	−0.7 (3)	C3—C2—S1—C9	−0.18 (16)
C18—C19—C20—C21	−179.7 (2)		

### Hydrogen-bond geometry (Å, º)

Cg is the centroid of the C5–C7/C9/N1/N2 ring.

**Table:**

*D*—H···*A*	*D*—H	H···*A*	*D*···*A*	*D*—H···*A*
C13—H13···O1^i^	0.93	2.60	3.343 (4)	138
C10—H10···*Cg*^ii^	0.93	2.61	3.513 (4)	147

Symmetry codes: (i) −*x*+1, −*y*+2, −*z*+1; (ii) −*x*−1, −*y*−1, −*z*−1.
